# Effects of Sled Towing on Peak Force, the Rate of Force Development and Sprint Performance During the Acceleration Phase

**DOI:** 10.1515/hukin-2015-0042

**Published:** 2015-07-10

**Authors:** María Asunción Martínez-Valencia, Salvador Romero-Arenas, José L.L. Elvira, José María González-Ravé, Fernando Navarro-Valdivielso, Pedro E. Alcaraz

**Affiliations:** 1 UCAM Research Center of High Performance Sport, San Antonio Catholic University of Murcia, Guadalupe, Murcia, Spain.; 2 Faculty of Sport Sciences, Department of Sport and Physical Activity, San Antonio Catholic University of Murcia, Guadalupe, Murcia, Spain.; 3 Sport Research Center, University Miguel Hernández, Elche, Spain.; 4 Sport Performance Lab, Faculty of Sport Science, University of Castilla La-Mancha, Toledo, Spain.

**Keywords:** load cell, resisted sprint, maximal running, speed training

## Abstract

Resisted sprint training is believed to increase strength specific to sprinting. Therefore, the knowledge of force output in these tasks is essential. The aim of this study was to analyze the effect of sled towing (10%, 15% and 20% of body mass (Bm)) on sprint performance and force production during the acceleration phase. Twenty-three young experienced sprinters (17 men and 6 women; men = 17.9 ± 3.3 years, 1.79 ± 0.06 m and 69.4 ± 6.1 kg; women = 17.2 ± 1.7 years, 1.65 ± 0.04 m and 56.6 ± 2.3 kg) performed four 30 m sprints from a crouch start. Sprint times in 20 and 30 m sprint, peak force (Fpeak), a peak rate of force development (RFDpeak) and time to RFD (TRFD) in first step were recorded. Repeated-measures ANOVA showed significant increases (p ≤ 0.001) in sprint times (20 and 30 m sprint) for each resisted condition as compared to the unloaded condition. The RFDpeak increased significantly when a load increased (3129.4 ± 894.6 N·s−1, p ≤ 0.05 and 3892.4 ± 1377.9 N·s−1, p ≤ 0.01). Otherwise, no significant increases were found in Fpeak and TRFD. The RFD determines the force that can be generated in the early phase of muscle contraction, and it has been considered a factor that influences performance of force-velocity tasks. The use of a load up to 20% Bm might provide a training stimulus in young sprinters to improve the RFDpeak during the sprint start, and thus, early acceleration.

## Introduction

In athletics, sprint velocity over an extended distance (60 m and 100 m) is divided into three different phases: acceleration, constant velocity (or maximal velocity), and deceleration. The ability to generate strong acceleration is an important determinant of success in individual sports such as track sprinting, as well as team-based sports including soccer, rugby, field hockey, or football (Bangsbo et al., 1991; Hay, 1993; Lockie et al., 2003). With specific training, athletes should be able to improve sprint performance in the different phases (Korchemny, 1985; Mero and Komi, 1994; Young et al., 1995).

Most of the training programs designed to improve sprint running incorporate maximal strength, plyometrics, and specific strength training (Cronin and Hansen, 2006; Delecluse, 1997). For example, it has been recommended that athletes use 1) traditional resistance training with heavy loads to develop strength at slow velocities; 2) power training with light loads and higher velocities to increases force output at higher velocities and the rate of force development (RFD); 3) plyometric training to improve stretch-shortening cycle performance increasing the overall neural stimulation of the muscle, and thus, force output; and 4) sport-specific technique training in order to advance specific skills and coordinate force application (Cormie et al., 2011b; Cronin and Hansen, 2006; Weyand et al., 2000). Although it is common sprint training practice to use maximal strength exercises, vertical jumps and their derivatives, it has been postulated that a more specific training method for sprinting would use horizontal force production in unilateral movement, such as sled towing or resisted sprinting (Harris et al., 2008; Zafeiridis et al., 2005).

Resisted sprinting involves the athlete sprinting with an added load, in an attempt to provide velocity and movement pattern specificity during power training for the acceleration phase of sprinting (Cronin and Hansen, 2006). Ideally, athletes using sled towing should imitate the range of motion, body position, muscle activation time, and sprint velocity near the running speeds used in competition (Alcaraz et al., 2009; Kanehisa and Miyashita, 1983; Kaneko et al., 1983; Murray et al., 2005). Furthermore, Okkonen and Häkkinen (2013) found that resisted sprint exercises produce greater muscular activation when compared to free sprinting. Additionally, this training method increases force output (Alcaraz et al., 2014; Harrison and Bourke, 2008), stride length in the acceleration phase in team sport athletes (Kawamori et al., 2014a), the maximum velocity phase in sprinters (Alcaraz et al., 2014), and stride frequency in the acceleration and maximum speed phase (Clark et al., 2010; Zafeiridis et al., 2005). Resisted sprint training exercises are expected to increase the athlete’s ability to generate horizontal and vertical forces during sprinting (Cottle et al., 2014; Kawamori et al., 2014b), depending on the direction of the applied resistance arising from the training exercise (Zatsiorsky, 1995). Therefore, adding an extra load in specific exercises can be an appropriate strategy to achieve this specificity in trained athletes (Alcaraz et al., 2009, 2014).

Several studies have examined the effects on kinematics during sprint bouts performed with sled towing (Lockie et al., 2003; Maulder et al., 2008; Murray et al., 2005). Collectively, these studies found that sprinting with a weighted sled reduced the athlete’s stride length, stride frequency and sprint performance, increased ground contact time and the forward lean of the trunk, as well as produced some changes in the configuration of the athlete’s lower limbs during the ground contact phase of the stride (a thigh, shank, hip and knee angle and angular velocity). The magnitudes of the effects were dependent on the weight added to the sled, and recommendations were proposed for a load to provide a training stimulus without inducing detrimental changes in sprinting technique (Alcaraz et al., 2008). The literature had focused on the percentage of loss in maximal velocity in a sled-towing exercise. In this sense, the recommendations were that the load should be sufficient to reduce speed to approximately 90% of maximal velocity (Letzelter, 1995; Lockie et al., 2003), considering the athlete’s sprint time as an indirect indicator of the intensity of the exercise (Alcaraz et al., 2008).

Lockie et al. (2003) submitted 12.6% Bm as a load that reduced maximal 15 m velocity by approximately 10% in field sport players, whereas Alcaraz et al. (2008) suggested 9.9% Bm in the maximum velocity phase in track athletes. Thus, a higher load for the acceleration phase has been suggested when compared to the maximum velocity phase, but the recommendations were determined according to the effects on sprint kinematics. In this regard, Maulder et al. (2008) proposed 10% Bm as the load to improve athlete’s sprint start and early acceleration performance in accordance with the lack of significant changes in running technique. Maulder et al. (2008) also suggested that training with approximately 20% Bm may produce some benefits, aside from the “negative” changes in technique. Otherwise, several studies had shown that resisted sprint training had minimal impact on lower-body kinematics during the acceleration (Clark et al., 2010; Lockie et al., 2013; Spinks et al., 2007) and maximum velocity (Alcaraz et al., 2014) phases. As can be seen, many studies set the load in sled-towing exercise to a percentage of the athlete’s body mass (Alcaraz et al., 2009; Cronin et al., 2008; Linthorne and Cooper, 2013; Lockie et al., 2003; Martínez-Valencia et al., 2014; Maulder et al., 2008; Murray et al., 2005), based on the fact that larger athletes tend to generate greater muscular power output (Martínez-Valencia et al., 2013). However, only two studies (Cottle et al., 2014; Kawamori et al., 2014b) have been found that analyzed the acute effects of the sled-towing exercise on ground reaction force, but the results were not conclusive.

According to the literature (Cronin and Sleivert, 2005; Young et al., 1995), maximal force production and the RFD are strongly related to tasks such as sprinting, where force production should range between 80 and 160 ms (Young et al., 1995). Additionally, Cormie et al. (2011b) suggested that an important consideration for improved athletic performance influenced the RFD, which relates to the ability to generate more force in shorter periods of time. Owing to the high relationship between the RFD and sprint performance, the aim of this study was to determine the effects on force-time traces and sprint performance when young athletes completed 30 m sprint towing a weighted sled with a load derived from a percentage of their body mass (0%, 10%, 15% and 20% Bm). It was hypothesized that sled-towing exercise in the early acceleration phase would increase the RFD when loads higher than 10% Bm were used, and time to reach the RFDpeak would increase when higher loads were used in young sprinters.

## Material and Methods

### Participants

Twenty-three participants (17 men and 6 women) were recruited for the study (men = 17.9 ± 3.3 years, 1.79 ± 0.06 m and 69.4 ± 6.1 kg; women = 17.2 ± 1.7 years, 1.65 ± 0.04 m and 56.6 ± 2.3 kg). The participants were active competitive track athletes who specialized in sprinting. Subjects had previous sled-towing training experience, and also at least two years of previous maximal strength training. As Alcaraz et al. (2008) found that sled towing led to similar decreases in sprint time and effects on sprint kinematics for men and women, subjects were combined into only one group for the purpose of further statistical analysis. The study was approved by the Human Subjects Ethics Committee of the Catholic University of San Antonio, the participants were informed of the protocol and procedures prior to their involvement, and written consent to participate was obtained. Signed, informed consent was also obtained from parents/guardians for those subjects who were under 18 years of age.

### Measures

In order to analyze the effects of sled towing using 10%, 15% and 20% Bm on sprint performance and force measures, a quasi-experimental cross-sectional design was used. The selection of loads was based on resistance similarly employed in the previous research (Alcaraz et al., 2008; Lockie et al., 2003, Maulder et al., 2008; Murray et al., 2005). The literature has used running velocity as indirect measure of the overload (Alcaraz et al., 2008), as well as the effects on sprinting kinematics, suggesting that velocity should not fall by more than 10% (Letzelter et al., 1995; Lockie et al., 2003). As a result, the present study set the load between 10% and 20% Bm to avoid a great loss of velocity, and not excessively influence sprinting technique.

### Procedures

**Figure 1:**
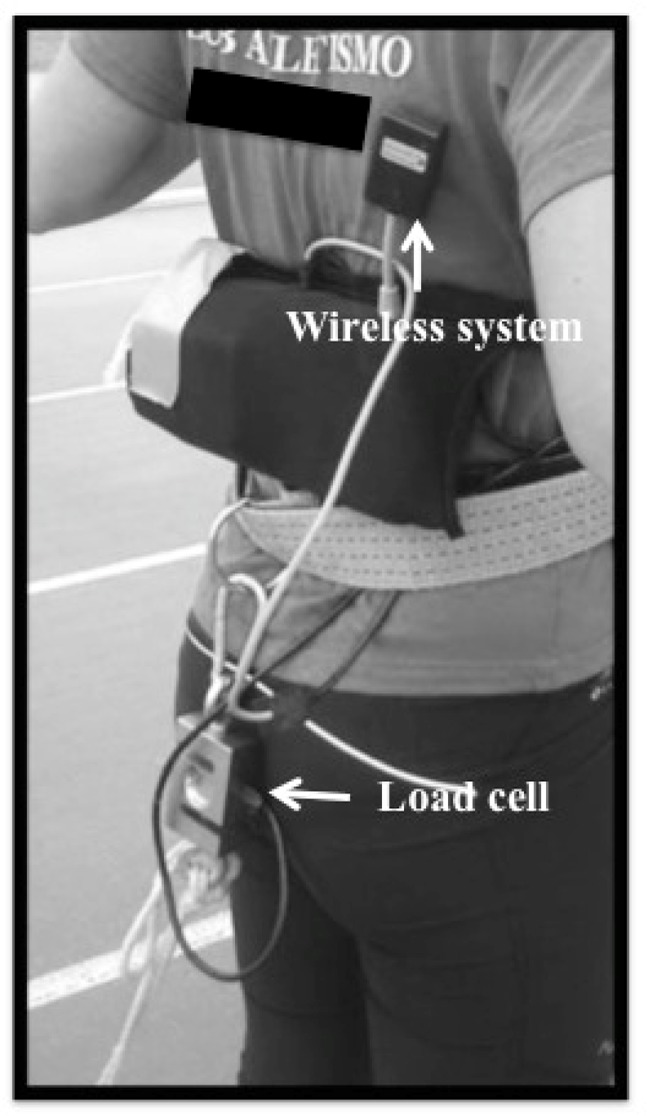
Load cell placement

Data were collected in one session. The sprint trials were conducted on an outdoor synthetic track. Testing was carried out during the pre-season phase of athlete’s training when the athletes were following maximal strength, resisted, acceleration, and maximum-velocity sprint training. The training program consisted of four sessions, and two maximal strength training sessions per week. Anthropometric information was collected prior to the warm-up. Body height and mass (Seca780, Vogel & Halke, Germany) were measured before starting the sprint trials to determine the loads relative to 10%, 15% and 20% Bm. Afterwards, the participants performed a specific warm-up consisting of 8 min of running, 8 min of active stretching, 10 min of running technique exercises, and 2–4 submaximal and maximal short sprints. The sprint trials were performed using a 4.7 kg weighted sled (Power Systems Inc., Knoxville, TN) attached to each athlete by a 3.6 m cord and waist harness. A load cell (MuscleLab, Ergotest Innovation, Norway) was attached between the waist harness and the cord (Figure 1). The load cell was calibrated by the use of standard loads and the signal analysis software (Musclelab 4000e, Ergotest Innovation). Participants wore their own athletic training clothes and spiked sprint shoes.

The athletes performed four 30 m sprints (unloaded sprints and sprints pulling resistances of 10%, 15%, and 20% of Bm) from a crouch start. One trial was assessed for each load. Considering the results of Hunter et al. (2004), horizontal velocity was the most reliable variable (CV = 0.4%; ICC = 0.99). Additionally, force measures (i.e. GRF) during the propulsive phase had been reported to present high reliability (CV = 1.9%; ICC = 0.96). Similarly, Lima et al. (2011) calculated peak and mean power using a load cell to record force measures in a semi-tethered test, and test-retest trials suggested the reliability of these measures. Furthermore, Murray et al. (2005) used two sets of sprints in their study, and the results revealed that the times in Test 2 were slower. As a result, Murray et al. (2005) suggested that one test would produce valid data. Previous studies that had analyzed the effect of sled towing on sprint performance also recorded one sprint for different load conditions (Cronin et al., 2008; Martínez-Valencia et al., 2013; Martínez-Valencia et al., 2014). As a result, this procedure was adopted in the current study. The participants began 1 m behind the starting line for each sprint. Before starting to run, the athletes were required to pull softly the sled to keep the cord and load cell with a uniform tension. The trial order was randomized for each participant, and a rest period of at least 6 min was given between trials to minimize the effects of fatigue on sprint performance.

**Figure 2:**
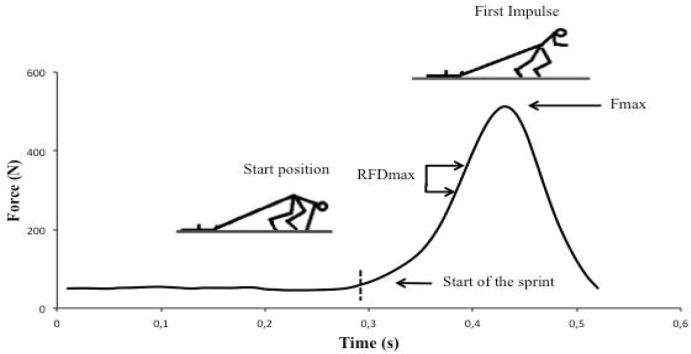
Force-time trace for start in sled-towing exercise, illustrating the start of the sprint, the RFD and Fpeak

In order to record the sprint times over 30 m, a photocell system (DSD Lasersystem, DSD S.L., Leon, Spain) was placed at 0 m, 20 m, and 30 m. The load cell recorded changes in force production during the first step, when the athlete started to run. The force-time traces for the sprints were analyzed to obtain three dependent variables: Fpeak, RFDpeak and TRFD, all in the first step. The RFDpeak corresponded to the steepest gradient of the force-time curve over a 0.02 s period (RFD = ΔF·Δt^−1^). The TRFD was obtained by finding the time difference between the start of the sprint and the beginning of the RFDpeak. The start of the sprint was defined as the point where the force reading was greater than the average force when the subject was static in the starting position (Figure 2).

### Statistical Analysis

Descriptive statistical methods were used to calculate mean and *SD*. A repeated-measures analysis of variance (ANOVA) with Bonferroni post hoc adjustments was used to determine whether there was a significant interaction between dependent variables under the various resisted conditions. Power (*1-β*) and effect sizes (η^2^p) were also calculated. The effect size was calculated with Partial Eta squared (η^2^p). All statistical analysis was computed using SPSS 20.0 for Mac OS X (IBM, New York, USA), and the level of significance was set at *p* ≤ 0.05.

## Results

**Figure 3:**
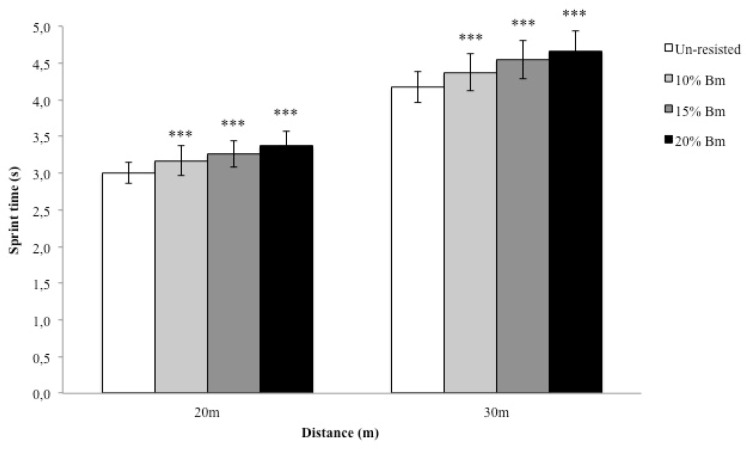
Increments in sprint time across all testing conditions in the 20 m and 30 m sprint *** = significant differences (p ≤ 0.001) from the unload sprint

The results showed that all increments in resistance resulted in significant increases in sprint time, both in the 20 m and 30 m sprints (Figure 3). The sprint times when towing a sled with a load of 10%, 15% and 20% Bm were significantly different from that of the un-loaded sprint for both the 20 m (*p* < 0.001; η^2^p = 0.909; *1-β* = 1.00) and 30 m (*p* < 0.001; η^2^p = 0.915; *1-β* = 1.00) intervals. In addition, significant differences (*p* < 0.05) were found between each resisted condition (10%, 15%, and 20% Bm) in both distances.

In line with the changes in sprint time, the increments in resistance induced significant increases in the RFDpeak (*p* < 0.05; η^2^p = 0.748; *1-β* = 0.827) (Table 1). The RFDpeak with 15%, and 20% Bm significantly rose when compared to the RFDpeak with 10% Bm (*p* < 0.05 and *p* < 0.01, respectively). The TRFD showed increments from 0.134 to 0.163 s when the load increased, but no significant differences were found (*p* = 0.95 and *p* = 0.10, respectively). As expected, peak force in the first step value was achieved with the 20% Bm load, but there were no significant changes in peak force when increasing resistance (Table 1).

## Discussion

The results showed that an increase in loads led to a decrease in sprint performance over 20 and 30 m when athletes ran under different conditions (10%, 15%, and 20% Bm). Similar results may be found in previous studies (Alcaraz et al., 2009; Cronin et al., 2008; Keogh et al., 2010; Letzelter et al., 2005; Linthorne, 2013; Lockie et al., 2003; Martínez-Valencia et al., 2014; Maulder et al., 2008; Murray et al., 2005). Lockie et al. (2003) found that loads of 12% and 32% Bm decreased velocity during 15 m acceleration to 91% and 76%, respectively. The results from the current study showed a sprint time increase of approximately 5% in 20 and 30 m sprint time with 10% Bm, and a 10% increase with 20% Bm. This is somewhat in contrast to Murray et al. (2005) who agreed that there was an increase in sprint time with an increase in resistance, but considered that the impact on sprint performance was not significant using resistance from 5% to 30% Bm.

The major finding of the present study was that the RFDpeak with 15% and 20% Bm significantly increased when compared to the 10% Bm condition. According to the results from this study, if the load is too light, the stimulus may not be sufficient to reach the RFDpeak during the acceleration phase. Maulder et al. (2008) also agreed that a load of approximately 10% Bm may not provide a sufficient training stimulus in a block start action. The use of higher loads for sled towing may provide training stimulus for young athletes, when also considering the increase in muscular activity in sled towing (Okkonen and Häkkinen, 2013). Cormie et al. (2011b) suggested the use of an optimal load would provide an effective stimulus to elicit increases in maximal power output. According to the impulse-momentum relationship (Keogh et al., 2010), Hunter et al. (2004) reported increases in sprinting speed occurred as a result of an increase in the propulsive forces and the RFD. Therefore, when considering the effects upon the RFDpeak, sled towing may be an appropriate method to improve the acceleration phase performance in young sprinters.

To explain the RFD changes during the loaded sprints, the works of Moir et al. (2005) and Swinton et al. (2012), who investigated weighted vertical jumps, were considered. Both studies compared the RFD during unloaded and loaded (30% and 60% 1RM back squat and 0%, 20%, 40% and 60% 1RM, respectively) static jumps. Collectively, these studies found that the mean RFD and power decreased when the load increased, while converse results were found in the present study. These differences may be explained due to: 1) the exercise used for measuring the RFD (a concentric squat jump vs. specific-resisted sprinting); and 2) the magnitude of the load used (20–60% 1RM vs. 10–20% Bm). On the other hand, only Cottle et al. (2014) analyzed the effect of sled towing on the RFD in GRF during a sprint start. Cottle et al. (2014) described that the RFD increased for the front leg with an external load of 20% Bm, but not for the back leg in field and court sport athletes. These differences when compared to the present results may be due to the different devices used to record force production in both studies. Cottle et al. (2014) recorded GRF via a force plate, while the current study recorded force while athletes towed the sled by the load cell. It should be noted that a limitation of the present study was that force-time traces were not recorded in the unloaded condition as the sled was not used, and thus the load cell could not be attached between the athlete and the sled. The present study highlights that sled towing increases the ability to rapidly develop force of the lower limb musculature during the acceleration phase in young sprinters.

The results suggested an increment in time needed to reach the RFD. Although there was no significant effect on TRFD, caution must be taken when considering this result. If we take into account the suggestion of Cronin and Sleivert (2005) and Young et al. (1995) that maximal force production and the RFD were strongly related to tasks such as sprinting, and force production should range between 80 and 160 ms (Young et al., 1995), the load set during sprint sled towing should allow for force to be produced over a limited period of time. The increase in TRFD could be a function of increments in ground contact time (Lockie et al., 2003), and the higher loads may have lengthened the contact time for the first step, and by extension time to reach the RFD. Further research on force production while towing heavier loads is required to confirm this statement. Nonetheless, the results from the current study showed that any changes to TRFD across the different load conditions were not significant.

Weyand et al. (2000) found that the ability to produce a high average vertical GRF in a short stance time was an advantage to achieving a higher maximum velocity. Several studies have shown the effect on GRF during resisted sprint starts (Cottle et al., 2014; Kawamori et al., 2014b). Nevertheless, neither Cottle et al. (2014), nor Kawamori et al. (2014b), found a significant increase in peak propulsive GRF. Again, more force variables (i.e. GRF, mechanical power) should be considered in order to set the most accurate load in sled-towing exercises. A wide selection of resistance exercises has been recommended to improve athletes’ muscular power affecting different components of explosive muscle action (Newton and Kraemer, 1994). Athletes should train with resistance that elicits appropriate mechanical power production (Cormie et al., 2011b; Wilson et al., 1993) to increase their explosive power output. Lima et al. (2011) measured mechanical power in field running using the semi-tethered method with 18% Bm in a 120 m effort, but did not record the optimal load for eliciting peak power. Andre et al. (2013) recorded peak power during resisted sprinting on a non-motorized treadmill, and showed that subjects achieved peak power with 35% Bm. The optimal load for eliciting peak power in resisted sled towing for acceleration development still needs to be confirmed. Nonetheless, the use of the RFD as a measure to control the effect of sled towing may be adequate considering that an important aspect to improve athletic performance is the ability to generate more force in shorter periods of time (Cormie et al., 2011b). Thus, the increase in the RFDpeak and force found in the present study suggests the use of weighted sled towing for improvement of young athletes’ force output during the acceleration phase of a maximal sprint.

Resisted training studies have shown that this training method could increase muscular force output so as to improve sprinting speed (Alcaraz et al., 2014; Harrison and Bourke, 2009; Lockie et al., 2012; Spinks et al, 2007). In this sense, the results showed that increases in resistance involve increment in the RFDpeak with no significant decrease in TRFD, thus may positively influence a young athlete’s sprint start. The evaluation of data obtained suggested that resistance of 15–20% Bm should be used in resisted sprint training during the acceleration phase. In addition to this, it should be noted that these results focused on the early acceleration phase in young track sprinters, and the load would likely have provided a greater stimulus to increase their force production during a specific exercise. It should be also taken into account that the results combined sexes, and no separated analysis between men and women was conducted. Future studies may focus on the analysis of power output during resisted sprint with weighted sled, and investigate male and female sprinters in isolation. Nevertheless, the present study highlights the benefits of using a load up to 20% Bm in sled towing during the acceleration phase in young sprinters.

## Figures and Tables

**Table 1:** Mean ± SD RFD_peak_, Time to RFD_peak_ and F_peak_ during the first step of the run in sled-towing exercise

**Variable**	**10% Bm**	**15% Bm**	**20% Bm**
**RFDpeak (N·s^−1^)**	2286.8 ± 719.4	3129.4 ± 894.6^*^	3892.4 ± 1377.9^**^
**TRFD (s)**	0.133 ± 0.044	0.150 ± 0.041	0.163 ± 0.055
**Fpeak(N)**	270.5 ± 101.4	327.1 ± 51.8	408.3 ± 97.8

Bm = Body mass; RFD = rate of force development; TRFD = time to the RFD; Fpeak = peak force.

* p< 0.05;

** p < 0.01.
